# Diverged Populations Admixture Bolsters Genetic Diversity of a New Island Dibbler ( *Parantechinus apicalis* ) Population, but Does Not Prevent Subsequent Loss of Genetic Variation

**DOI:** 10.1111/eva.70073

**Published:** 2025-01-21

**Authors:** Rujiporn Thavornkanlapachai, Harriet R. Mills, Kym Ottewell, Cathy Lambert, J. Anthony Friend, Daniel J. White, Zahra Aisya, W. Jason Kennington

**Affiliations:** ^1^ School of Biological Sciences The University of Western Australia Crawley Western Australia Australia; ^2^ Department of Biodiversity, Conservation and Attractions Biodiversity and Conservation Science Bentley Western Australia Australia; ^3^ School of Science Edith Cowan University Mount Lawley Western Australia Australia; ^4^ Department of Biodiversity, Conservation and Attractions Biodiversity and Conservation Science Albany Western Australia Australia; ^5^ Bennelongia Environmental Consultants Jolimont Western Australia Australia

**Keywords:** body size, captive breeding, genetic erosion, intraspecific hybridization, mate choice, translocation

## Abstract

Translocating individuals from multiple source populations is one way to bolster genetic variation and avoid inbreeding in newly established populations. However, mixing isolated populations, especially from islands, can potentially lead to outbreeding depression and/or assortative mating, which may limit interbreeding between source populations. Here, we investigated genetic consequences of mixing individuals from two island populations of the dibbler ( *Parantechinus apicalis* ) in an island translocation. Despite a high level of genetic divergence between the source populations (*F*_ST_ ranges 0.33–0.64), and significant differences in body size, individuals with different ancestries were able to successfully interbreed in captivity and in the wild. However, the genetic contributions from each source population were unequal initially despite each of the source populations contributing an equal number of founders. Mating success of captive animals based on the pedigree suggests that this bias toward one source population was due to founder mortality and the mating success of younger and heavier animals. Nevertheless, genetic contributions in the translocated population became equal over time with no parental purebreds, suggesting an extreme excess of hybrids across multiple years. While genetic variation in the translocated population was comparable or higher than the source populations, the increase was short‐lived. Genetic composition of captive animals may not reflect what happens in the wild. These changes post‐translocation highlight the need for continued genetic monitoring.

## 1 Introduction

Establishing new populations is an effective management tool for reducing the extinction risk to endangered species restricted to a few remnant populations (Johnson et al. 2010; Maguire and Lacy 1990) or populations that are threatened by introduced predators or diseases (Huxtable et al. 2015; Ottewell et al. 2014). However, because translocations are often based on small numbers of individuals, they can be prone to founder effects, which reduce genetic variation and lead to rapid genetic divergence from source populations (Broders et al. 1999; Gautschi et al. 2002; Ramstad et al. 2013). In threatened or endangered species, the choice of source populations is also frequently limited. Some may have low genetic diversity, high levels of inbreeding, or reduced fitness as the result of inbreeding depression (Eldridge et al. 1999; Grueber et al. 2010). Survivorship differences among founders and their offspring can further reduce the effective population size and subsequently exacerbate the loss of genetic diversity (Biebach and Keller 2012; Jamieson 2011). Loss of genetic variation is of particular concern as it reduces the evolutionary potential of a new population to cope with adverse changes and puts the population at risk of extinction (Eldridge et al. 1999; Frankham 1996; Willi, Van Buskirk, and Hoffmann 2006).

One way to counterbalance the loss of genetic diversity when establishing new populations is to use multiple source populations (Weeks et al. 2011). Indeed, several studies have shown that new populations established using founders from multiple source populations have higher genetic variation relative to one or more of the source populations (Kennington, Hevroy, and Johnson 2012; Ransler, Quinn, and Oyler‐McCance 2011; Thavornkanlapachai, Mills, et al. 2019; White et al. 2018). While there are clear advantages to using multiple source populations when establishing a new population, there are potential costs as well. Intrinsic (environment independent) and extrinsic (environment dependent) incompatibilities between populations can reduce fitness in hybrid and backcrossed offspring (Allendorf et al. 2001; Kulmuni and Westram 2017; Lynch 1991; Rhymer and Simberloff 1996). In addition, differences in mating behavior and mate recognition may lead to pre‐zygotic reproductive barriers between source populations (Rolán‐Alvarez et al. 1999; Vines and Schluter 2006). There may be survivorship differences among founders from different source populations and their offspring due to maladaptation to the release site (Brodie 1992; Campbell and Waser 2001) or post‐zygotic barriers (Álvarez and Garcia‐Vazquez 2011; Arntzen et al. 2009). All these factors can reduce the effective population size of a newly established population and subsequently lead to loss of genetic variation and inbreeding (Frankham 1995).

The dibbler ( *Parantechinus apicalis* ) is a small (approximately 40–125 g) marsupial with a generalist and opportunistic diet, consisting mainly of insects (Miller et al. 2003). They are semi‐arboreal and crepuscular. There is strong sexual dimorphism, with males being larger than females (Mills and Bencini 2000; Mills, Moro, and Spencer 2004). Females breed once a year during autumn (mid‐April to May) and can produce as many as eight young per breeding season (Mills 2004). These young reach sexual maturity after 10–11 months (Woolley 1995). The mating system is likely to be polygynandrous as females were observed to mate with multiple males in captivity (Lambert and Mills 2006) and this is a common system for dasyurid species (Holleley et al. 2006; Kraaijeveld‐Smit, Ward, and Temple‐Smith 2002). In the wild, dibblers can live up to 4 years (Friend and Collins 2005). In captivity, they can live up to 5.5 years (Lambert pers. comm.).

**FIGURE 1:**
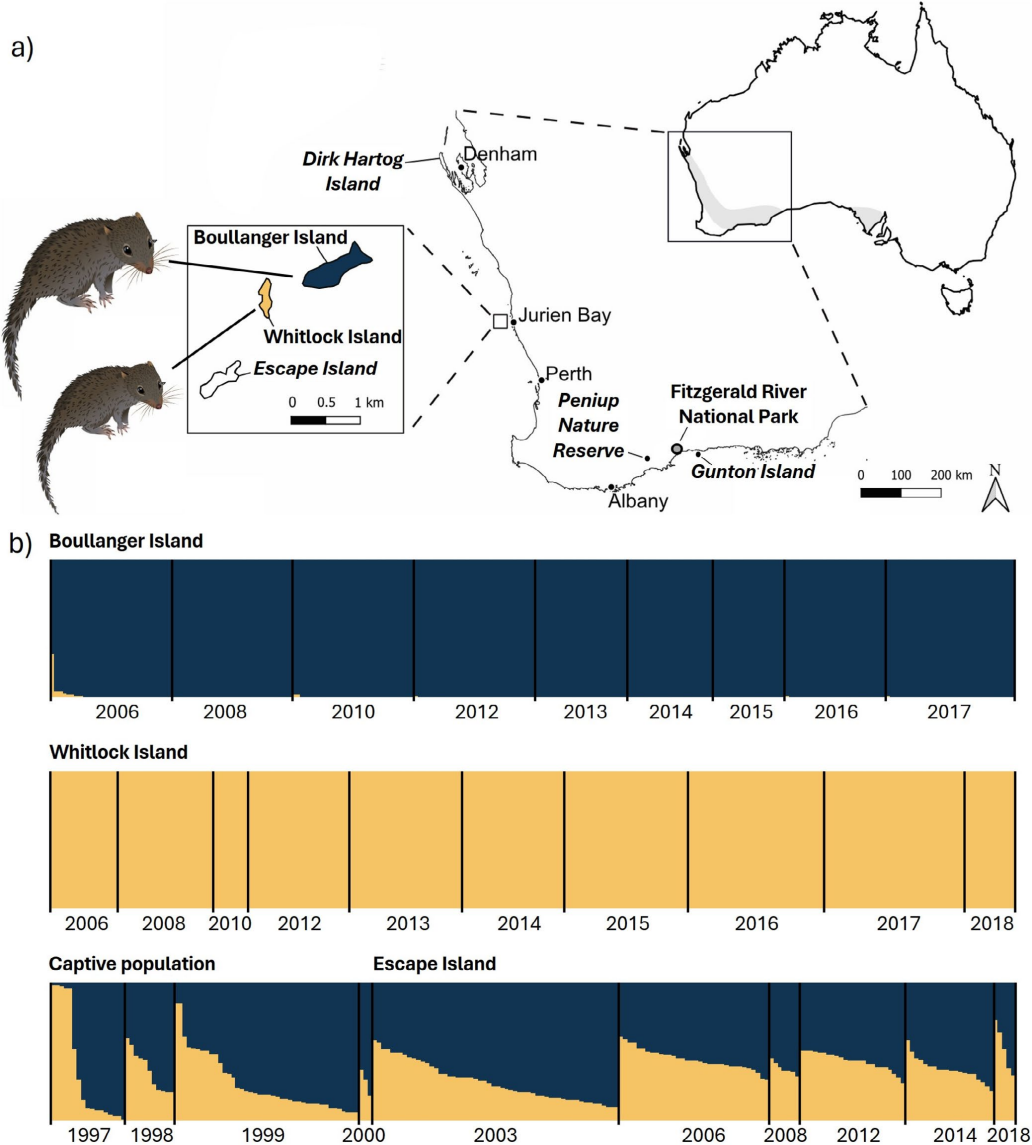
(a) Current and historical distribution of the dibbler ( *Parantechinus apicalis* ) adapted from Aisya et al. (2022). The past distribution is highlighted in gray on the Australian continent on the top right. The current natural populations are in bold font and translocated populations are in italic bold font. Dibbler image indicates body size differences between two islands. (b) Summary of the clustering analysis on the source (Boullanger and Whitlock Islands), captive, and translocated population on Escape Island assuming two admixed populations (*K* = 2). Each individual is represented by a bar showing the individual's estimated membership to a particular cluster (represented by different colors). Black lines separate samples collected over different years from each of the populations. Samples post 2012 collections are from Aisya et al. (2022). The dibbler image was sourced from Creazilla under an Attribution 4.0 International (CC BY 4.0) license. Image can be found at https://creazilla.com/nodes/64031‐dibbler‐clipart.

The dibbler is endemic to the southwest of Australia (Miller et al. 2003; Mills and Bencini 2000; Mills, Moro, and Spencer 2004; Thavornkanlapachai, Kennington, et al. 2019; Woolley 2008). The species is listed as Endangered under the Environment Protection and Biodiversity Conservation Act (1999) and the IUCN Red List of Threatened Species (Burbidge and Woinarski 2016). *P. apicalis* were once widely distributed in Western Australia (WA) from Shark Bay on the central west coast to Esperance on the southern coastline and east to the Eyre Peninsula, South Australia (Baynes 1987, 1990; Friend 2003; Figure 1a). However, due to threats from introduced predators, such as foxes ( *Vulpes vulpes* ) and feral cats ( *Felis catus* ), inappropriate fire regimes, habitat degradation by dieback (*Phytophthora cinnamomi*), and competition with house mice ( *Mus musculus* ) on islands (Friend 2003; Northover et al. 2022), they now only occur naturally in the Fitzgerald River National Park (~300,000 ha) on the southern coast of WA, and on two small islands, Boullanger and Whitlock Islands off the mid‐west coast of WA (Fuller and Burbidge 1987; Morcombe 1967). Currently, < 1000 mature individuals are estimated to be on the mainland (Burbidge and Woinarski 2016; Woinarski, Burbidge, and Harrison 2014) and approximately 100 individuals are known to be alive on both islands (Moro 2003). In order to provide founder stock for translocation to new sites, a captive breeding program at Perth Zoo was established in 1998 (Friend 2003). There are several translocations to mainland reserves and islands established from the captive‐bred population at Perth Zoo (Friend 2003; Northover et al. 2022). Only translocations to the Peniup proposed nature reserve (Thavornkanlapachai et al. 2021), Escape Island (Aisya et al. 2022; Moro 2003), Gunton Island (Northover et al. 2022), and Dirk Hartog Island (Northover et al. 2022) have been successful so far.

Boullanger and Whitlock Islands have been separated from mainland Australia for at least 500 years (Chalmers and Davies 1984). Dibblers on the mainland have higher genetic diversity and are genetically diverged from the island dibblers (Mills, Moro, and Spencer 2004). They are also larger, live longer, and have earlier oestrus and a longer gestation period (Friend and Collins 2005; Mills et al. 2012; Mills, Moro, and Spencer 2004). As a result of these phenotypic and trait differences, the island populations have been managed and captive‐bred separately from the mainland population. The island populations are also genetically differentiated from each other (Aisya et al. 2022; Mills, Moro, and Spencer 2004). While both island populations have low genetic diversity relative to the mainland, the Boullanger Island population has higher genetic diversity and a lower level of inbreeding than the Whitlock Island population (Mills, Moro, and Spencer 2004). In addition, male dibblers on Boullanger Island are slightly heavier and larger than those on Whitlock Island (Mills and Bencini 2000; Mills, Moro, and Spencer 2004). Female dibblers on Boullanger Island also produce a higher number of pouch young on average (Mills, Moro, and Spencer 2004). On the islands, males exhibit facultative die‐off, dying after the first mating season in some years but not in all years (Mills and Bencini 2000). This happens more frequently on Boullanger Island than Whitlock Island (Mills and Bencini 2000), thought to be associated with differing levels of nutrient inputs from seabirds (Wolfe et al. 2004). No facultative male die‐off has been observed in mainland populations or in captivity (Friend and Collins 2005; Lambert and Mills 2006; Wolfe et al. 2004).

The translocation to Escape Island was the first to use founders cross‐bred from both natural island populations. While the translocation appears to have been successful and no facultative male die‐off was observed during the early phase of the translocation (Moro 2003), it is unclear if or how well the source population lineages have introgressed and whether genetic variation within the Escape Island population has changed over time. The aim of this study is to investigate the genetic changes taking place during the breeding phase within the captive population at Perth Zoo and in the subsequent translocated population on Escape Island over a 22‐year period. Our specific aims were to (i) determine the extent of mixing between the source population lineages and to assess whether levels of genetic variation within the translocated and source populations have changed over time, (ii) investigate factors influencing mating success in captivity, and (iii) study effects of genetic mixing on body measurements in comparison to the source populations.

## 2 Methods

### 2.1 Captive Breeding Program

To support a translocation to Escape Island, a captive‐breeding colony was set up at Perth Zoo using 11 broodstock founders, two pairs from Boullanger Island, and two pairs from Whitlock Island in 1996 and three additional males from Whitlock Island added in 1999. Males and females with the lowest mean kinship value were paired together. In cases where a female showed aggression or no mating behaviors toward a selected male, the next male on the list would be introduced until all females were allocated a partner. Further details on husbandry, pairing design, and breeding behaviors of island *P. apicalis* are described in Lambert and Mills (2006) and Wolfe, Robertson, and Bencini (2000). A total of 33 adults and 55 subadults (51 females and 37 males) were sequentially released on Escape Island in 1998 (26 animals), 1999 (41 animals), 2000 (19 animals), and 2001 (2 animals). Eighty‐three of these animals were captive‐bred and five animals were broodstock founders. More details on the translocation can be found in Moro (2003). The pedigree of this captive‐bred population is detailed in Table S1.

### 2.2 Sampling and DNA Extraction

Samples used in this study were obtained from the captive breeding program or were collected during monitoring of *P. apicalis* populations on Escape (10.5 ha), Boullanger (26 ha), and Whitlock (5.4 ha) Islands carried out by the Western Australian Department of Biodiversity, Conservation and Attractions (DBCA). The trapping on Boullanger and Whitlock Islands took place between 2006 and 2013 in May and October while limited accessibility to Escape Island only permitted trapping in December, January, and February. All wild‐born and captive‐bred dibblers had either an ear tissue or hair sample taken and a microchip implanted. Wild‐born dibblers had their body measured for head length, pes width and length, tail width and length, scrotum width and length, then weighed and their reproductive status recorded. Captive‐bred dibblers were weighed and had age recorded during mating seasons. All ear tissue and hair samples were stored in a 20% DMSO solution saturated with NaCl at room temperature or 70% ethanol at −80°C. DNA from 65 ear notch samples was extracted using a salting‐out method (Sunnucks and Hales 1996) with the following modifications: DNA was incubated at 56°C rather than 37°C and 10 mg/mL instead of 0.1 mg/mL Proteinase K was added to 300 μL solution of TNES. DNA from 14 hair samples (one from year 1997 and 13 from year 1999) was extracted using BioBasic ONE 4 ALL Genomic DNA Miniprep Kit, following the protocol described in the instruction manual except DNA was eluted in 30 μL of Buffer CE instead of 50 μL. DNA was extracted from a maximum of 30 samples per collection year in each source population. In total, DNA was extracted from 76 samples from the captive population (1997, *N* = 19; 1998, *N* = 11; 1999, *N* = 43; 2000, *N* = 3), 120 tissue samples from Boullanger Island (2006, *N* = 30; 2008, *N* = 30; 2010, *N* = 30; and 2012, *N* = 30), 53 from Whitlock Island (2006, *N* = 12; 2008, *N* = 17; 2010, *N* = 6; and 2012, *N* = 18), and 137 samples from Escape Island (2002, *N* = 15; 2003, *N* = 47; 2006, *N* = 44; 2008, *N* = 7; and 2012, *N* = 24).

### 2.3 Microsatellite Variation

Genotypes were determined at 14 out of 28 microsatellite loci screened (3.1.2, 3.3.1, 3.3.2, 4.4.2, 4.4.10, pPa2A12, pPa7A1, pPa1B1O, pPa2B1O, pPa4B3, pPa2D4, pDG1A1, Sh6e, and Aa4A) that were characterized from *P. apicalis* and closely related species (Table S2). Polymerase chain reaction (PCR) was performed in a 10 μL reaction volume using the QIAGEN Multiplex PCR Kit with primer concentrations ranging from 0.04 to 1.5 μM and from 10 to 20 ng of DNA (Table S2). Amplifications were performed on an Eppendorf Mastercycler epgradientS Thermocycler using the following parameters: 15 min at 95°C, a total of 35 or 40 cycles of 30 s at 94°C, 90 s at the specific annealing temperature in Table S2, 60 s at 72°C, and concluding with 30 min at 60°C. PCR products were analyzed on an ABI 3730 sequencer using a GeneScan‐600 LIZ internal size standard and scored using GENEMARKER version 1.90 (SoftGenetics). Due to the low volume and yield from DNA extraction, only one PCR per multiplex was carried out for each hair sample. Genotyping success of hair samples was 54.6%, and genotypes were screened for unusual alleles. In addition, approximately 10% of tissue samples were reamplified to calculate genotyping error rates.

### 2.4 Data Analysis

To assess genotype quality, we calculated the allele‐ and locus‐specific genotypic error rates (Pompanon et al. 2005). Individuals who failed in DNA extraction and with < 3 successful genotypes were excluded from further analysis. Escape Island 2002 and 2003 cohorts were trapped in December and March, respectively, so samples from 2002 were merged into the 2003 March collection for further analysis. The rest of the year collections were trapped within a single year. Broodstock founders of the captive population were also removed from analyses. The final numbers of samples used in analyses are summarized in Table 1. With our sample collections from early years, we were able to extend temporal analyses of more recent samples collected from all islands from Aisya et al. (2022). Genotypes from Aisya et al. (2022) were standardized across all loci to ensure consistency of allelic calls between the two data sets. We tested for the presence of null alleles in the source populations at each locus using MICROCHECKER (Van Oosterhout et al. 2004). In all subsequent analyses, only temporal/population samples with at least five individuals were used. Estimates of genetic variability within population samples were assessed by calculating the allelic richness (an estimate of the number of alleles per locus corrected for sample size) and gene diversity using the FSTAT software package (Goudet 2001). Deviations from Hardy–Weinberg Equilibrium (HWE) were quantified using the inbreeding coefficient (*F*_IS_) and tested for significance using randomization tests. Positive *F*_IS_ values indicate deficiency of heterozygotes, relative to random mating, whereas negative values indicate a heterozygote excess. Genotypic disequilibrium between each pair of loci within each population sample was assessed by testing the significance of association between genotypes. Genetic differentiation between pairs of population samples was assessed using Weir and Cockerham's (1984) *F*_ST_ (θ). Estimates of *F*_IS_, pairwise *F*_ST_ values, tests for differentiation among samples, deficits in heterozygotes, and genotypic disequilibrium were calculated using the FSTAT software package (Goudet 2001). A sequential Bonferroni correction (Rice 1989) was applied to all tests to control for type I statistical errors arising from multiple testing. Differences in estimates of genetic variation and *F*_IS_ values between population samples were tested using Wilcoxon's signed‐rank tests with samples paired by locus using the stats package in the R version 4.3.0 (R Core Team 2023).

**TABLE 1:** Genetic variation estimated from 14 microsatellite loci within the source, captive, and translocated populations of *Parantechinus apicalis* .

Sample	*N*	*H*	AR	*P*	*F*_IS_	GD	BP	Reference
Source population: Boullanger Island								
2006	30	0.40 ± 0.05	1.39 ± 0.05	1.00	0.06	0	0.98 ± 0.01	This study
2008	30	0.35 ± 0.06	1.35 ± 0.06	0.86	−0.07	1	1.00 ± 0.00	This study
2010	30	0.34 ± 0.06	1.34 ± 0.06	0.86	0.08	1	1.00 ± 0.00	This study
2012	30	0.33 ± 0.07	1.33 ± 0.07	0.71	−0.01	1	1.00 ± 0.00	This study
2013	23	0.33 ± 0.06	1.32 ± 0.06	0.71	0.06	1	1.00 ± 0.00	Aisya et al. (2022)
2014	21	0.30 ± 0.07	1.30 ± 0.07	0.64	−0.06	1	1.00 ± 0.00	Aisya et al. (2022)
2015	18	0.27 ± 0.06	1.27 ± 0.06	0.64	0.04	1	1.00 ± 0.00	Aisya et al. (2022)
2016	25	0.30 ± 0.07	1.30 ± 0.07	0.64	−0.02	1	1.00 ± 0.00	Aisya et al. (2022)
2017	32	0.28 ± 0.06	1.28 ± 0.06	0.64	−0.04	1	1.00 ± 0.00	Aisya et al. (2022)
Source population: Whitlock Island								
2006	12	0.18 ± 0.07	1.17 ± 0.07	0.36	**0.45**	0	0.00 ± 0.00	This study
2008	17	0.15 ± 0.06	1.15 ± 0.06	0.43	0.01	0	0.00 ± 0.01	This study
2010	6	0.18 ± 0.06	1.17 ± 0.06	0.43	0.31	0	0.00 ± 0.02	This study
2012	18	0.06 ± 0.03	1.06 ± 0.03	0.21	0.25	0	0.00 ± 0.03	This study
2013	20	0.05 ± 0.03	1.05 ± 0.03	0.14	0.03	0	0.00 ± 0.04	Aisya et al. (2022)
2014	18	0.04 ± 0.03	1.04 ± 0.03	0.21	0.05	0	0.00 ± 0.05	Aisya et al. (2022)
2015	22	0.05 ± 0.03	1.05 ± 0.03	0.21	0.14	0	0.00 ± 0.06	Aisya et al. (2022)
2016	24	0.04 ± 0.03	1.04 ± 0.03	0.14	−0.03	0	0.00 ± 0.07	Aisya et al. (2022)
2017	25	0.05 ± 0.03	1.05 ± 0.03	0.14	−0.04	0	0.00 ± 0.08	Aisya et al. (2022)
2018	9	0.02 ± 0.02	1.02 ± 0.02	0.07	−0.14	0	0.00 ± 0.09	Aisya et al. (2022)
Captive population								
1997	17	0.40 ± 0.06	1.40 ± 0.06	0.93	0.17	3	0.63 ± 0.10	This study
1998	11	0.40 ± 0.08	1.34 ± 0.08	0.71	−0.04	0	0.64 ± 0.04	This study
1999	42	0.39 ± 0.05	1.39 ± 0.05	0.93	−0.06	5	0.73 ± 0.03	This study
2000	3	—	—	—	—	—	0.72 ± 0.06	This study
Translocated population: Escape Island								
2003	56	0.41 ± 0.05	1.41 ± 0.05	0.93	0.04	2	0.72 ± 0.02	This study
2006	34	0.44 ± 0.05	1.43 ± 0.05	0.93	**0.26**	0	0.56 ± 0.01	This study
2008	7	0.36 ± 0.06	1.36 ± 0.06	0.79	−0.24	0	0.62 ± 0.02	This study
2012	24	0.38 ± 0.06	1.38 ± 0.06	0.79	0.04	1	0.57 ± 0.01	This study
2014	20	0.39 ± 0.05	1.39 ± 0.05	0.86	−0.07	1	0.63 ± 0.02	Aisya et al. (2022)
2018	5	0.40 ± 0.06	1.40 ± 0.05	0.86	0.08	0	0.48 ± 0.08	Aisya et al. (2022)

*Note: N* is the sample size, *H* is gene diversity, AR is allelic richness, *P* is the percentage of polymorphic loci, *F*_IS_ is the inbreeding coefficient, GD is the number of pairs of loci in genotypic disequilibrium, and *BP* is the average estimated genetic membership to Boullanger Island ancestry. *F*_IS_ values significantly greater than zero (*p* value < 0.05) are highlighted in bold text. Standard errors are given after mean values. Collection years with sample size < 5 are not included in the analyses.

To investigate the extent of genetic mixing between source population lineages in the translocated population on Escape Island, we carried out Bayesian clustering analysis using the software package STRUCTURE 2.3.4 (Pritchard, Stephens, and Donnelly 2000). The analyses were performed with the source population locations set as prior information and the number of genetic clusters (*K*) set to two because this was the number of source populations used to establish the captive and translocated populations. We used admixture and independent allele frequency models as indicated in Mills, Moro, and Spencer (2004) since Boullanger and Whitlock Island populations are genetically divergent with extremely rare to no gene flow between these populations. Individuals were assigned a membership coefficient, which is the fraction of the genome with membership to a particular cluster. Ten independent runs were performed using 100,000 iterations, with a burn‐in period of 10,000 iterations. These parameter values yielded highly consistent results across independent runs, indicating the number of iterations and burn‐in period were sufficiently long. The STRUCTURE estimated cluster membership coefficient over multiple runs were permuted using the Greedy option to obtain a mean across replicates in CLUMPP (Jakobsson and Rosenberg 2007). The output from CLUMPP was depicted in R (R Core Team 2023). We cross‐checked the *Q*‐value results with the pedigrees of captive animals to ensure the estimated contribution/s is consistent with the given known pairings. Pedigree records were consistent with STRUCTURE estimates indicating a reliable estimate for those on Escape Island.

We obtained the captive population pedigree and mating behavior observation and carried out a generalized linear model (GLM) analysis to investigate factors influencing mating success (a male successfully mated with a female) and reproductive outcomes (number of offspring and proportion of offspring surviving). These factors included parental weight and age, and the ancestry differences between the mating pair (scored as 1 or 0 for the same or different source population, respectively). As some individuals bred over multiple years, we entered individual identity (stud number) as a random intercept in all models. These analyses were carried out using the glmmTMB version 1.1.10 (Brooks et al. 2017), MuMIn version 1.48.4 (Barton 2024), car version 3.1 (Fox and Weisberg 2019), DHARMa version 0.4.7 (Hartig 2024), and RVAideMemoire version 0.9‐38‐7 (Hartig 2024; Herve 2023) packages in R (R Core Team 2023).

To test if phenotypic differences between source populations have a genetic basis, genetic proportions of Boullanger Island ancestry and various body measurements of dibblers born in captivity and on Escape Island were tested using Spearman's rank correlation. Differences in body measurements between wild‐born adults on different islands were compared for each sex using Kruskal–Wallis rank sum tests and then Wilcoxon's signed‐rank tests for post hoc analysis. All analyses were carried out using R (R Core Team 2023).

## 3 Results

### 3.1 Genetic Variation Within Populations

Across this study and the Aisya et al. (2022) genetic data set, we had a successful amplification rate of 0.922 ± 0.008 and 0.995 ± 0.002 per locus respectively. The allele‐specific and locus‐specific genotypic error rates from this study averaged across all loci were 0.004 ± 0.003 and 0.008 ± 0.006 respectively. Three loci were identified as having null alleles (3.3.2, pPa7A1, and Sh6e) within population samples. Because there was no consistent pattern in the presence of null alleles (i.e., the loci with null alleles varied among samples from the same population and between populations), all loci were retained for further analysis. Overall estimates of genetic diversity in the captive and translocated populations were higher than the source populations (Table 1). Estimates of gene diversity and allelic richness were significantly higher in the translocated Escape Island population than the Whitlock Island source population in 55 and 56 out of 60 pairwise tests, respectively (Wilcoxon rank‐sum tests, *p* < 0.05). However, there were no significant differences in gene diversity and allelic richness between the translocated and Boullanger Island populations in all comparisons. Temporal variations in allelic richness and gene diversity were observed between collection years in the Boullanger and Whitlock Island populations, but not in the translocated population (allelic richness: *𝜒* ^2^ = 26.1 and 34.3, *p* = 0.001 and *p* < 0.001; gene diversity: *𝜒* ^2^ = 27.8 and 34.3, both *p* < 0.001, respectively). There was also a trend of decreasing gene diversity and allelic richness in all populations, with many loci becoming monomorphic, especially the Whitlock Island population (Table 1).

Multilocus *F*_IS_ values fluctuated between years both in the source and the translocated populations but were significantly different from zero in only a few cases (Table 1). The Whitlock population had the highest overall multilocus *F*_IS_ value and was significantly different from zero (randomization tests, *p* < 0.002). The number of pairs of loci in genotypic disequilibrium (GD) ranged from one to five, with the highest level occurring in 1999 samples from the captive population. The number of pairs of loci in GD in the source population samples ranged from zero to one (Table 1).

### 3.2 Population Structure and Genetic Mixing Within the Translocated Population

**FIGURE 2:**
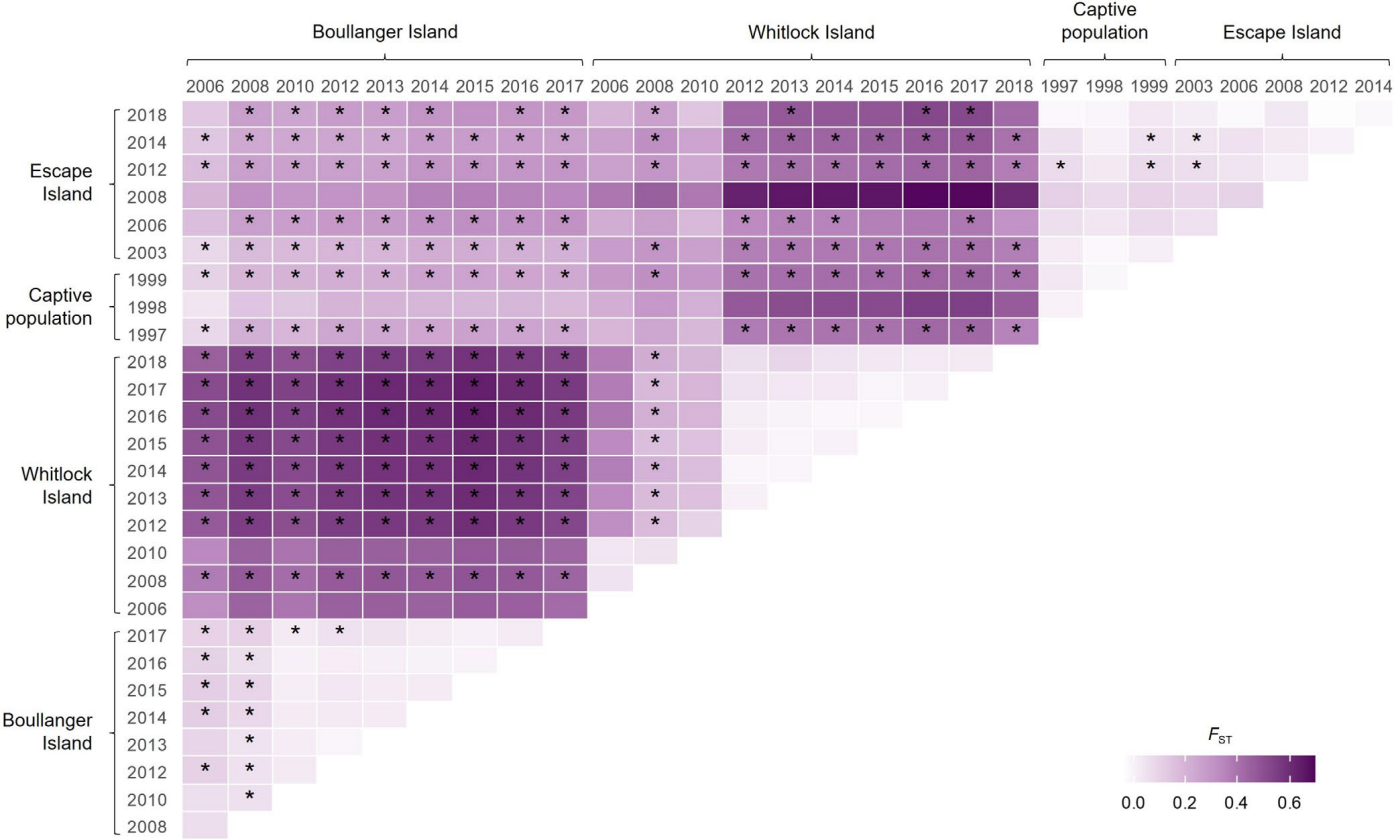
Pairwise *F*_ST_ estimates between source, captive, and translocated populations of *Parantechinus apicalis* . *F*_ST_ estimates significantly greater than zero after correction for multiple comparisons are marked in asterisks as followed *p < 0.000132, **p < 0.000026, and ***p < 0.000003.

Pairwise population *F*_ST_ indicated a substantial differentiation in allele frequencies between the source populations (Figure 2). There were also significant divergences between the translocated and source populations and between population samples taken in different years from the translocated population. Overall, *F*_ST_ values indicated the captive population and earlier collection years of the translocated population were more similar to the Boullanger Island population. However, the translocated population became more genetically diverged from both source populations with increasing *F*_ST_ values in recent collections (Figure 2).

The clustering analyses also revealed clear genetic differences between the source populations and changes in the genetic composition of the translocated population over time. Individuals from Boullanger Island were predominantly assigned to one genetic cluster and those from Whitlock Island were assigned to the other (Figure 1b). Most individuals from the captive and translocated populations had membership to both genetic clusters, indicating they had mixed ancestry (Figure 1b). Overall, individuals within the captive and translocated populations had a higher proportion of membership to the genetic cluster associated with Boullanger Island, though this pattern changed over time as the proportion of membership between the two genetic clusters became more even in the translocated population (Figure 1b and Table 1).

The bias toward the genetic cluster associated with Boullanger Island in the captive and translocated populations was consistent with the pedigree record. Across a total of 83 dibblers born in captivity, based on pedigree information, 85.2% of them had a strong Boullanger Island ancestry (pure‐bred Boullanger Island and first or second generation backcross to Boullanger Island). The remaining 14.8% of individuals were F_1_ hybrids or backcrosses to Whitlock Island.

### 3.3 Factors Influencing Mating and Reproductive Success in Captivity

The generalized linear models revealed that mating success in captivity was associated with female weight and female age with the model coefficients indicating that heavier and younger females had higher mating success (Table 2). No other factor had an effect on mating success and none of the factors examined, including whether or not the offspring had mixed ancestry (i.e., parents had the same or different ancestries), were significantly associated with the number of offspring or the proportion of offspring that survived (Table 2).

**TABLE 2:** Results of generalized linear models investigating factors influencing mating and reproductive success of *Parantechinus apicalis* in captivity.

Factor	*b*	SE	*z*	*p*
Mating success				
**Female weight**	**3.69**	**1.81**	**2.03**	**0.042**
**Female age**	**−50.61**	**25.39**	**−1.99**	**0.046**
Male weight	2.92	1.72	1.70	0.090
Male age	−9.24	7.25	−1.27	0.202
Mate ancestry	−32.48	30.15	−1.08	0.281
Number of offspring				
Female weight	0.02	0.02	0.969	0.333
Female age	0.04	0.22	0.200	0.841
Male weight	0.00	0.01	−0.059	0.953
Male age	−0.16	0.23	−0.72	0.471
Mate ancestry	−0.12	0.32	−0.377	0.706

*Note:* The table shows coefficient values (*b*), standard errors (SE), *z* values, and significant *p* values. Significant factors are highlighted in bold text.

### 3.4 Morphology of Genetic Admixed Island Dibblers in Captivity and in the Wild

**FIGURE 3:**
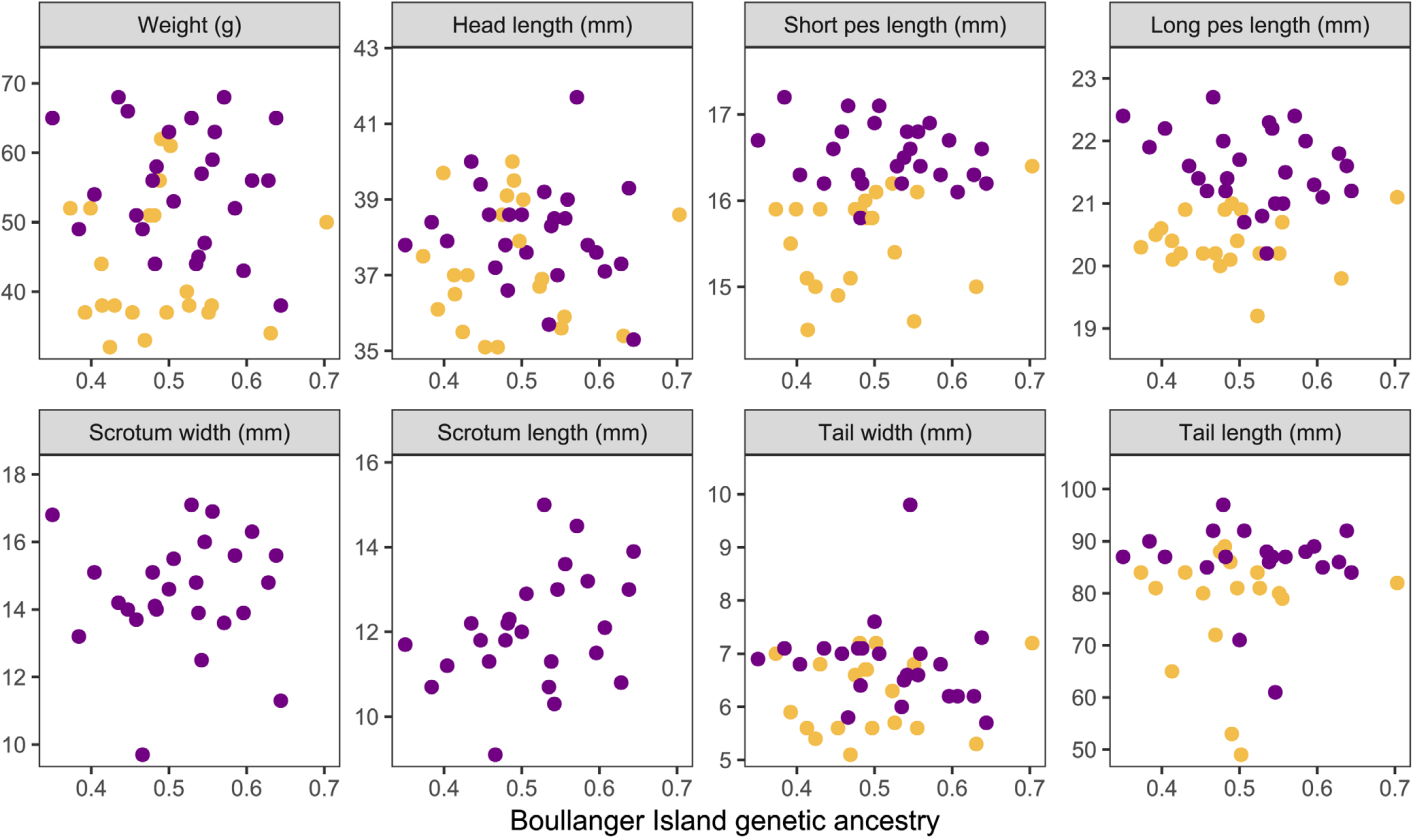
The relationship between Boullanger Island ancestral genetic proportions and body measurements of adult male (purple) and female (gold) *Parantechinus apicalis* captured on Escape Island between 2006 and 2012.

**FIGURE 4:**
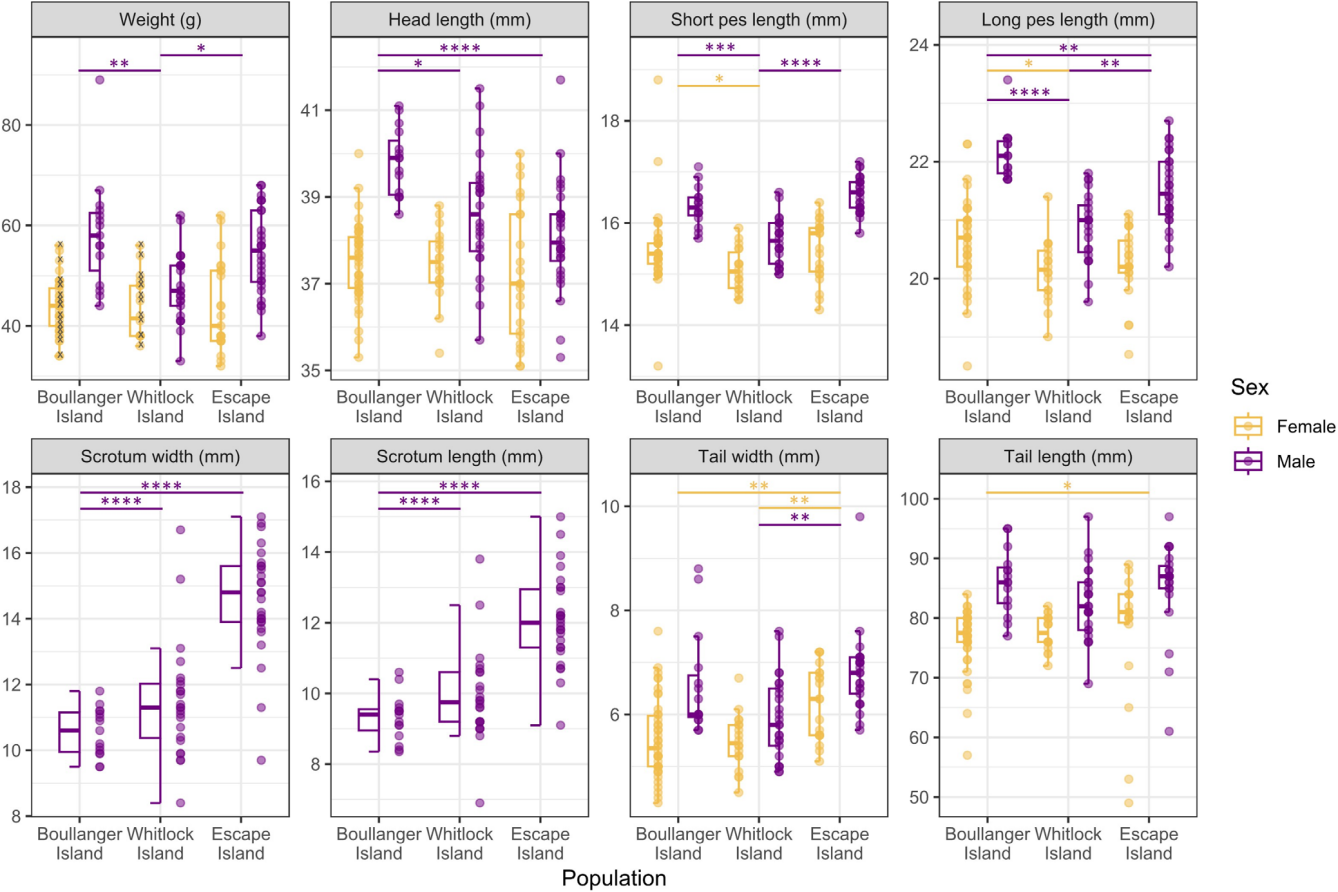
Body measurements of adult *Parantechinus apicalis* males (purple) and females (gold) captured on Boullanger Island, Whitlock Island, and Escape Island between 2006 and 2013. Escape Island population was trapped at a different time of the year due to accessibility. The enlarged scrotum of Escape Island males was coincided with spermatorrhea periods. Crosses on female body weight indicate the measurement was taken while pouch young were carried. Pairwise Wilcoxon's signed‐rank test significances are indicated as asterisks at levels of *p < 0.05, **p < 0.01, ***p < 0.001, and ****p < 0.0001.

There were strong positive correlations between the genetic proportion of Boullanger Island ancestry and body weight of captive‐born dibblers in both sexes (females: *ρ =* 0.738, *S =* 403, *p* < 0.001; males: *ρ* = 0.797, *S* = 11, *p =* 0.032, Figure S1). However, these positive correlations were not observed in the wild‐born dibblers on Escape Island in both sexes despite measurements from different years being analyzed separately (except for scrotum length in males, *ρ* = 0.400, *S* = 1561, *p =* 0.048; Figure 3, Figures S2 and S3). At the population level, males on Boullanger Island were significantly larger than males on Whitlock Island (except for tail width and length, Figure 4). Interestingly, males on Whitlock Island had larger scrotums than males on Boullanger Island (Figure 4). Females showed no body size differences between Boullanger and Whitlock Island populations except Boullanger Island females had larger pes (Figure 4). Females on Boullanger Island were more likely to have a full litter of eight young than Whitlock Island females, but there were no significant differences between average numbers of pouch young (Escape Island 7.0 ± 0.24 0.1, *N* = 35, Moro (2003); Boullanger Island: 6.97 ± 0.24, *N* = 34; Whitlock Island: 6.35 ± 0.36, *N* = 17; *W* = 372, *p* = 0.082, Figure S4). Body size differences between admixed individuals on Escape Island and purebred individuals on Boullanger and Whitlock Islands were more pronounced in males than females. Males on Escape Island were more similar to males on Boullanger Island on many traits (short pes length and tail size) though Escape Island males had a smaller but similar head length to Whitlock Island males (Figure 4). While males on Escape Island had a much larger scrotum than the other islands, they were measured at a different time of the year and were enlarged as the result of spermatorrhea (Figure 4). Females on Escape Island had wider and longer tails, but other body measurements remained similar to females on Boullanger and Whitlock Islands (Figure 4).

## 4 Discussion

### 4.1 Genetic Divergences Between Source Populations and Consequences of Genetic Mixing

Differential mortality and variance in reproductive success can have profound effects on the genetic composition of newly established populations (Biebach and Keller 2012; Hynes et al. 2005; Miller et al. 2010; Nelson et al. 2002; Pope et al. 2012). Previous studies have shown that mortality can reduce the number of founders by as much as half (e.g., *Capra ibex* , Biebach and Keller 2012 and *Sphenodon guntheri* , Nelson et al. 2002), while mating preferences arising from male to male competition or female mate choice (Hynes et al. 2005; Miller et al. 2010; Pope et al. 2012) can disproportionately favor certain individuals or source populations. In this study, we were able to show that despite substantial genetic and body size differences between source populations, individuals from different island populations were able to mate and produce viable offspring with mixed ancestries, both in captivity and in the wild. Furthermore, admixture continued to occur over multiple generations at the translocation site. However, the genetic contributions of source populations in the captive‐bred colony differed from that observed in the wild translocated population.

The initial genetic composition of the captive‐bred colony was more similar to the Boullanger Island ancestry while the wild translocated population showed an equal mix between the two island ancestries in later years. Mating behavior observation in captivity suggests that males with a Boullanger Island ancestry may have a reproductive advantage over males with a Whitlock Island ancestry due to their larger size. Consistent with this, 12 of 14 mating attempts of Whitlock Island males with captive females failed. The bias was also exacerbated by the mortality of one female from Whitlock Island before the first mating season. Despite the initial bias toward the Boullanger Island ancestry, the wild translocated population showed all individuals with evenly admixed ancestry and a complete lack of parental types, suggesting an extreme excess of hybrids across multiple years. It is unclear the mechanism underlying such a pattern of hybrid excess. Strong disassortative mating could drive the production and maintenance of hybrids. For example, studies on a closely related species, *Antechinus agilis* , have shown that a male's mating success is influenced by genetic compatibility where females preferred genetically dissimilar males, whereas males mated readily with most females (Parrott et al. 2015). Alternatively, admixed individuals may exhibit strong heterosis as the result of adaptive introgression, whereby an increase in fitness occurs via the insertion of new gene variants into the population (Harrison and Larson 2014), or through increased genome‐wide heterozygosity providing relief from inbreeding depression due to masking of genetic load (Dussex et al. 2023). A previous study suggests that Escape Island males have a similar body size and females have a similar average number of pouch young as Boullanger Island animals (Moro 2003) suggesting similar fitness outcomes, at least in their home environments. The absence of both pure parental types on Escape Island could imply the presence of recessive deleterious alleles in either source population that are lethal or highly maladaptive in the novel Escape Island environment when exposed in homozygous form but are masked in hybrid individuals. Further research would be required to unravel the contribution of these mechanisms to maintaining hybrid excess on Escape Island.

Translocated populations often experience a significant loss of genetic variation and become genetically distinct from their source populations as a result of founder effects, variance in the reproductive success of founders and genetic drift (Biebach and Keller 2009; Broders et al. 1999; Gautschi et al. 2002; Hundertmark and Van Daele 2010; Mock, Latch, and Rhodes 2004). However, our study showed that the translocated population on Escape Island had higher or similar levels of genetic variation compared with the source populations, consistent with previous studies involving multiple source populations (Kennington, Hevroy, and Johnson 2012; Ransler, Quinn, and Oyler‐McCance 2011; Stockwell, Mulvey, and Vinyard 1996; Thavornkanlapachai, Mills, et al. 2019; White et al. 2018). The increase in genetic diversity in the translocated population may be attributed to several factors. First, admixture from multiple source populations is likely to have bolstered genetic variation in the translocated population (Huff, Miller, and Vondracek 2010; Kennington, Hevroy, and Johnson 2012; Ransler, Quinn, and Oyler‐McCance 2011; Stockwell, Mulvey, and Vinyard 1996). Second, large *P. apicalis* litter size may aid a rapid population growth (Moro 2003) and minimize genetic diversity loss from random genetic drift (e.g., Deyoung et al. 2003). Last, multiple releases of captive‐bred individuals may have replenished genetic diversity lost when the translocated population was established (Williams and Scribner 2010).

Our analysis of morphological variation in the captive and wild translocated populations indicated that body size differences between source populations had an underlying genetic basis. This was evident from the positive relationship between the genetic ancestry of admixed individuals and variation in multiple traits, specifically in relation to male size. The morphological differences of animals on different islands could reflect adaptation to local environments or differences in genetic variation. The larger area of Boullanger Island may favor larger males who can travel a greater distance for mates (e.g., Jenkins et al. 2007; Lomolino 2005; McNab 2002). A previous investigation on *P. apicalis* male die‐off phenomenon found a link between male die‐off and resource availability on Boullanger and Whitlock Islands (Wolfe et al. 2004). The frequency of male die‐off on different islands was negatively correlated to the presence of nesting large seabirds, especially on Whitlock Island, which provides soil nutrients and improves plant productivity and subsequent invertebrate prey availability. A positive correlation between heterozygosity and fitness‐related traits could play a role in the larger body size and mating success of Boullanger Island males in captivity (e.g., Annavi et al. 2014). Last, males on Escape Island were similar in size to males on Boullanger Island in multiple traits (short pes length and tail width and length). However, some traits were intermediate between the two island populations (long pes length) or smaller, but similar to the smaller‐sized ancestry (head length). Such deviations could be driven by factors such as levels of genetic diversity, heterosis, local adaptation, or non‐additive genetic effects (Carlborg and Haley 2004; Stockwell and Weeks 1999). No facultative male die‐off was observed in Escape Island population despite having the dominant Boullanger Island genetic ancestry in early generations (Moro 2003).

### 4.2 Temporal Variation in the Genetic Composition of Island Populations

Despite the initial increase of genetic variation in the translocated population, levels of genetic variation in the source populations declined over time in parallel with the changes observed in population size. Population viability analysis suggested that drought had the greatest impact on the survival of *P. apicalis* populations (Aisya et al. 2022). The *P. apicalis* capture rates on both Boullanger and Whitlock Islands have fluctuated over time but were declining and the captured numbers remained low on both islands since 2017 (Northover et al. 2022). Population recovery was indicated by the high proportion of subadults captured on both islands after a significantly higher rainfall over 2021 winter (J.A. Friend, unpubl. data). The large fluctuation of capture rates likely contributes to the small effective population size noted for island dibbler populations (Aisya et al. 2022), suggesting that all populations are likely to be vulnerable to high levels of genetic drift. Consistent with this expectation, both source and translocated populations exhibit increased numbers of monomorphic loci and reduced genetic variation in recent collection years. In addition, there was evidence of genetic differentiation with increased *F*_ST_ values between the source and translocated populations. Lack of genetic diversity limits population's capacity to adapt to changes. It is particularly a concern for future captive breeding programs and translocations sourcing from these island populations as the Whitlock Island population showed the greatest loss of genetic diversity over the course of this study.

### 4.3 Management Implications

Newly established populations are prone to loss of genetic variation, genetic drift, and inbreeding (Frankham 1995). Our study shows that using multiple source populations can increase genetic diversity within a newly established population. However, the increase is not much more than the most diverse source population. Population viability analysis of island populations showed that without interventions, Boullanger, Whitlock, and Escape Island populations will be extinct within 50, 34, and 35 years, respectively (Aisya et al. 2022). There have been some interventions by removing or reducing threats to the island populations such as house mice removal (Northover et al. 2022). However, due to limited carry capacity and isolation, all island populations continue to have a low effective population size (*N*_e_) and experience population fluctuations (Aisya et al. 2022 and Northover et al. 2022). The lack of gene flow between populations, in combination with population fluctuations, means genetic diversity will continue to decline as shown in this study and Aisya et al. (2022). Genetic diversity within each population will continue to decline at a rate of $\frac{1}{2N_{e}}$ per generation (Allendorf 1986), the smaller *N*_e_, the greater loss. The loss of genetic diversity over time would be more pronounced in the number of polymorphic loci as heterozygosity is a less sensitive measure to genetic loss (Allendorf, Hössjer, and Ryman 2024). One way to slow down the loss and increase *N*_e_ is to manage these island populations as a meta‐population and facilitate gene flow between each of the remnant island populations (Hanski and Gaggiotti 2004). Alternatively, facilitating gene flow from the mainland population could theoretically provide a greater increase in genetic diversity due to the greater allelic diversity of the mainland population and greater divergence in allele frequencies compared to the island populations. In the absence of experimental outcomes of interbreeding of mainland and island populations, a safer recommendation would be to establish new populations using island populations to increase global census size (*N*_c_) and *N*_e_ and manage all islands as a meta‐population. For future captive and translocation programs, we recommend taking animal's weight and age into pairing consideration to improve mating success and using multiple source populations to maximize genetic diversity.

## Conflicts of Interest

The authors declare no conflicts of interest.

## Supporting information


Data S1.

## Data Availability

Genotypes from all analyzed samples are stored in a database at the Department of Biodiversity, Conservation and Attractions and are available upon request. The additional analyses from this study are available in the Supporting Information.
