# Supplementary material for: Diverged Populations Admixture Bolsters Genetic Diversity of a New Island Dibbler ( Parantechinus apicalis ) Population, but Does Not Prevent Subsequent Loss of Genetic Variation
Source: Evol Appl. 2025 Jan 21;18(1):e70073. doi: 10.1111/eva.70073 (PMC11750805; doi:10.1111/eva.70073)
Supplement: Supplementary file 1 — Data S1. [file EVA-18-e70073-s001.docx]

**Supplementary information**

**Table S1** Pedigrees of the released *Parantechinus apicalis* on Escape Island between 1997 and 2000. Labels: wild-born Boullanger Island (BB), captive-born purebred Boullanger Island (BP), captive-born backcross Boullanger Island (BBC), Hybrid (H), captive-born purebred Whitlock Island (WP), and wild-born Whitlock Island (WW).

| Year | Female ID | Male ID | Survive | Die | Total | Observation |
| --- | --- | --- | --- | --- | --- | --- |
| 1997 | BB4 | BB1 | 6 | 0 | 6 |  |
| 1997 | WW8 | BB2 | 7 | 0 | 7 |  |
| 1997 | BB3 | BB2 | 6 | 2 | 8 |  |
| 1998 | H29 | BB1 | 0 | 0 | 0 | No mating behaviour |
| 1998 | BP9 | BB1 | 0 | 0 | 0 | No mating |
| 1998 | BB3 | BB2 | 0 | 0 | 0 | No mating behaviour |
| 1998 | H17 | WW7 and BP24* | 0 | 0 | 0 | No mating |
| 1998 | BP25 | BB1 | 4 | 1 | 5 |  |
| 1998 | H16 | WW7 and BP24* | 0 | 5 | 5 |  |
| 1998 | BP13 | BP24 | 0 | 6 | 6 |  |
| 1998 | BP9 | H19 and BP21 | 7 | 0 | 7 |  |
| 1998 | BP10 | H19 and BP21 | 0 | 8 | 8 |  |
| 1998 | BP23 | H15 | 5 | 3 | 8 |  |
| 1998 | BP22 | H15 | 8 | 1 | 9 |  |
| 1999 | BBC34 | WW79 | 0 | 0 | 0 | No mating, female aggression |
| 1999 | BP11 | WW7 | 0 | 0 | 0 | No mating, female aggression |
| 1999 | BP14 | BB1 | 0 | 0 | 0 | No mating, female aggression |
| 1999 | BP22 | H15 | 0 | 0 | 0 | No mating, female aggression |
| 1999 | BP22 | WW7 | 0 | 0 | 0 | No mating behaviour |
| 1999 | BP22 | WW78 | 0 | 0 | 0 | No mating, female aggression |
| 1999 | BP22 | WW79 | 0 | 0 | 0 | No mating, female aggression |
| 1999 | BP40 | WW7 | 0 | 0 | 0 | No mating, female aggression |
| 1999 | BP40 | WW78 | 0 | 0 | 0 | No mating, female aggression |
| 1999 | BP40 | WW79 | 0 | 0 | 0 | No mating, female aggression |
| 1999 | BP40 | WW80 | 0 | 0 | 0 | No mating, female aggression |
| 1999 | BP60 | BB1 | 0 | 0 | 0 | Mated but no young |
| 1999 | BP60 | WW6 | 0 | 0 | 0 | No mating behaviour |
| 1999 | H18 | WW78 | 0 | 0 | 0 | No mating, female aggression |
| 1999 | BBC34 | WW80 | 6 | 0 | 6 |  |
| 1999 | BP11 | BBC37 | 7 | 0 | 7 |  |
| 1999 | BP9 | BP21 | 7 | 0 | 7 |  |
| 1999 | BP14 | BP39 | 6 | 2 | 8 |  |
| 1999 | BP40 | BP57 | 8 | 0 | 8 |  |
| 1999 | BP41 | H15 | 8 | 0 | 8 |  |
| 1999 | H18 | BB80 | 0 | 8 | 8 |  |
| 2000 | BP11 | H15 | 2 | 0 | 2 |  |
| 2000 | BP9 | BP21 | 2** | 1 | 3 |  |

* Both males were introduced to the female in the same breeding season

**One was transferred interstate

Five animals survived but never left and died in captivity

**Table S2:** Characteristics of the 28 microsatellite loci that were trialed, selected, and optimized for use in characterizing the genetic variability of the dibbler, *Parantechinus apicalis*.

| **Locus** | **Species** | **Nature of repeat** | **Trial Anneal Temp (C°)** | **Size (bp)** | **Multiplex** | **Cycle** | **DNA (10ng/uL)** | **PCR Anneal Temp (C°)** | **Primer sequence (5' - 3')** | **References** |
| --- | --- | --- | --- | --- | --- | --- | --- | --- | --- | --- |
| pPa2D4 | *P. apicalis* | NA | 60 | 193-197 | 2 | x35 | 1 | 56 | CAATCTGTCAATAACCTTCCCCC | Mills and Spencer 2003 |
|  |  |  |  |  |  |  |  |  | TGGAGGACCTCCAGAAAGTTAGC |  |
| pPa2A12 | *P. apicalis* | (GT)21 | 57 | 129-131 | 2 | x35 | 1 | 56 | ATCCTGGAGAAGAGAAGACCTGC | Mills and Spencer 2003 |
|  |  |  |  |  |  |  |  |  | GTGGCTTATTCCATGCTTGTAGG |  |
| pPa2B10 | *P. apicalis* | (GT)23 | 57 | 176-186 | 3 | x40 | 2 | 58 | GAGAAAAAATATGCACAAGCACC | Mills and Spencer 2003 |
|  |  |  |  |  |  |  |  |  | AAGGAGAAAAAGTTAATACCATCCC |  |
| pPa2D2 | *P. apicalis* | NA | Fail | NA |  |  |  |  | CAGAAAGAATAGAGTCCCATCACACA | Mills and Spencer 2003 |
|  |  |  |  |  |  |  |  |  | ATGGCCAATTTTAACTTTGTTGTTTA |  |
| pPa7A1 | *P. apicalis* | (GAA)85 | 57 | 298-315 | 2 | x35 | 1 | 56 | CTCCACCTCTCTAGACATGACCC | Mills and Spencer 2003 |
|  |  |  |  |  |  |  |  |  | TTTACTTGCTTTGTACTAGAGGCC |  |
| pPa7H9 | *P. apicalis* | NA | 56-57 | 166 | 2 | x35 | 1 | 56 | AAATAACAACAATAGTTCATTATGT | Mills and Spencer 2003 |
|  |  |  |  |  |  |  |  |  | ATTATTTGCTTACTTTGAAGATATA |  |
| pPa9D2 | *P. apicalis* | (GT)4AT(GT)11GC(GT)3 | 57 | 106 |  |  |  |  | TGGAAAGCAATATGGTAGAAGTGTG  TTCAAGGGTTCAAAACAACATTCTT | Mills and Spencer 2003 |
| pPa1B10 | *P. apicalis* | (GAAA)46 | 57-58 | 220-310 | 2 | x35 | 1 | 56 | AAGGAGGGATGGAGGAGGAA | Mills and Spencer 2003 |
|  |  |  |  |  |  |  |  |  | CAGTGTTCGAATGACATTGGCTAC |  |
| pPa4B3 | *P. apicalis* | (GT)15 | 57-58 | 121 |  |  |  |  | GAAGGACAACATTCCCGATTGT | Mills and Spencer 2003 |
|  |  |  |  |  |  |  |  |  | CCTACCCTAATTGCAAATCCTTTC |  |
| pPa8F10 | *P. apicalis* | (AC)19 | 57-58 | 100 |  |  |  |  | CAATCTAGGAATCACAGAACTCCC | Mills and Spencer 2003 |
|  |  |  |  |  |  |  |  |  | TTTGCATCTACCTAATTGCGTGT |  |
| pDG1A1 | *Dasyurus geoffroii* | (AG)20 | 54-60 | 190 |  |  |  |  | ATTTGCTTCTTGCTCCCTACAGC | Spencer et al., 2007 |
|  |  |  |  |  |  |  |  |  | TTTCACTCCTTCTGAGTTTATCACC |  |
| pDG1H3 | *Dasyurus geoffroii* | (TG)17 | 54-60 | 192-193 | 3 | x40 | 2 | 58 | GTGGATTGACACAATCAGAGTGG | Spencer et al., 2007 |
|  |  |  |  |  |  |  |  |  | GCAATTCCATCTTTATTGCATGC |  |
| pDg5G4 | *Dasyurus geoffroii* | (AC)24 | 60 | 545-549 | 3 | x40 | 2 | 58 | TAGATTCCTTCAATGGCTATCCC | Spencer et al., 2007 |
|  |  |  |  |  |  |  |  |  | GCTCCTGACATAGAGTGATGATGG |  |
| pDG6D5 | *Dasyurus geoffroii* | (AC)22 | 54 | 100 |  |  |  |  | CCTCCAGACAAATGCAACC | Spencer et al., 2007 |
|  |  |  |  |  |  |  |  |  | TCTCTGAATTTACTGATAGTATCTTTGG |  |
| pDG7F3 | *Dasyurus geoffroii* | (GT)24 | 46-48 | 170 |  |  |  |  | TCAGTTCAGCTACAACTGCTTGG | Spencer et al., 2007 |
|  |  |  |  |  |  |  |  |  | TGTTACATAGAGCATGAGCGACC |  |
| 3.1.2 | *Dasyurus spp.* | (CA)18 | 46-54 | 180 |  |  |  |  | AGGAAACTTCACAAGTGTCGA | Firestone, 1999 |
|  |  |  |  |  |  |  |  |  | ATTAATGACTCATCTGTTGTTGG |  |
| 3.3.1 | *Dasyurus spp.* | (CA)20 | 54-60 | 130 |  |  |  |  | CAGCCCTTGAGTCTTGAGATT | Firestone, 1999 |
|  |  |  |  |  |  |  |  |  | CATACCACCCCAGGAGTTTC |  |
| 3.3.2 | *Dasyurus spp.* | (CA)21 | 46-48 | 158-191 | 1 | x35 | 2 | 46 | AATAGCAGAGACTCGATCC | Firestone, 1999 |
|  |  |  |  |  |  |  |  |  | AGCCTTTATTACCTGGGAAG |  |
| 4.4.2 | *Dasyurus spp.* | (CA)19 | 54-58 | 127-129 | 2 | x35 | 1 | 56 | GAAATCCAAGCTCATTTTAG | Firestone, 1999 |
|  |  |  |  |  |  |  |  |  | AATCAACTCTGGAATGCATC |  |
| 4.4.10 | *Dasyurus spp.* | (CA)29 | 54-60 | 221-231 | 3 | x40 | 2 | 58 | AATGCTAGATTTCACTCCC | Firestone, 1999 |
|  |  |  |  |  |  |  |  |  | CCTCACATTTCTGGAACTG |  |
| 1.3 | *Dasyurus spp.* | (CA)15 | Fail | NA |  |  |  |  | ATTGATGAACAAGACATAGCG | Firestone, 1999 |
|  |  |  |  |  |  |  |  |  | TCATATAAGTCTTACTGTGCA |  |
| Sh2v | *Sarcophilus laniarius* | (AC)23 | Fail | NA |  |  |  |  | TTGGAGAAAATGGAAGCAG | Jones et al., 2003 |
|  |  |  |  |  |  |  |  |  | CAGGATCTATTTTCTGAGTTAAGG |  |
| Sh3o | *Sarcophilus laniarius* | (CA)22 | 46-50 | 194-196 | 1 | x35 | 2 | 46 | CTCAATGCCAAAGGTATCTTC | Jones et al., 2003 |
|  |  |  |  |  |  |  |  |  | CATAGTTCCAAATCACTCTCCAG |  |
| Sh6e | *Sarcophilus laniarius* | (CA)6 (A)2(CA)18 | 54-60 | 175-182 | 3 | x40 | 2 | 58 | GATTCTAGAAGGGATAGCAAGC | Jones et al., 2003 |
|  |  |  |  |  |  |  |  |  | GACACTCCATAGAAATGCACTG |  |
| Aa4A | *Antechinus agilis* | NA | 46-52 | 165-167 | 1 | x35 | 2 | 46 | TTTGATCCTCAGAGACTTGAT | Banks et al., 2005 |
|  |  |  |  |  |  |  |  |  | CCAAATCTACGTAAAATATCC |  |
| Aa4J | *Antechinus agilis* | NA | 46 | 166-178 | 1 | x35 | 2 | 46 | TCTTCAGTCTCTCAATGAGTT | Kraaijeveld-Smit et al., 2002 |
|  |  |  |  |  |  |  |  |  | AGAACACTCTAACAACATCCT |  |
| Aa7H | *Antechinus agilis* | NA | Fail | NA |  |  |  |  | AATTCAGTTGAGTCCACTTTG | Banks et al., 2005 |
|  |  |  |  |  |  |  |  |  | GTGCTTTCTCTGTCTTTCC |  |
| Aa7O | *Antechinus agilis* | NA | Fail | NA |  |  |  |  | GTCTTTGGATAATTGAAGTCTG | Kraaijeveld-smit et al., 2002 |
|  |  |  |  |  |  |  |  |  | GAATGAGGATCTAAGTGAATGT |  |

*
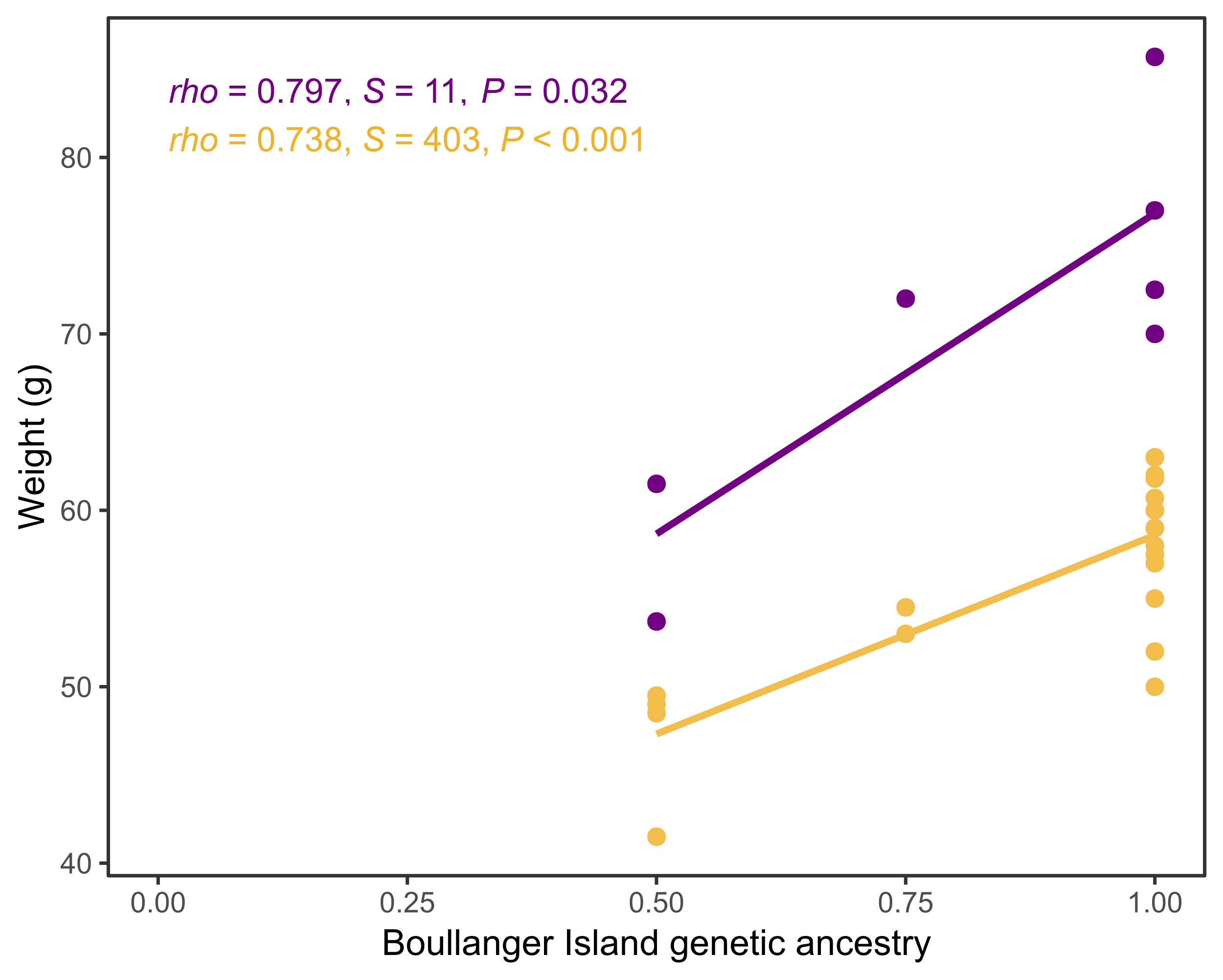
*

**Figure S1** The relationship between Boullanger Island ancestral genetic proportions and body weight of adult male (purple) and female (gold) *P. apicalis* born in captivity.

*
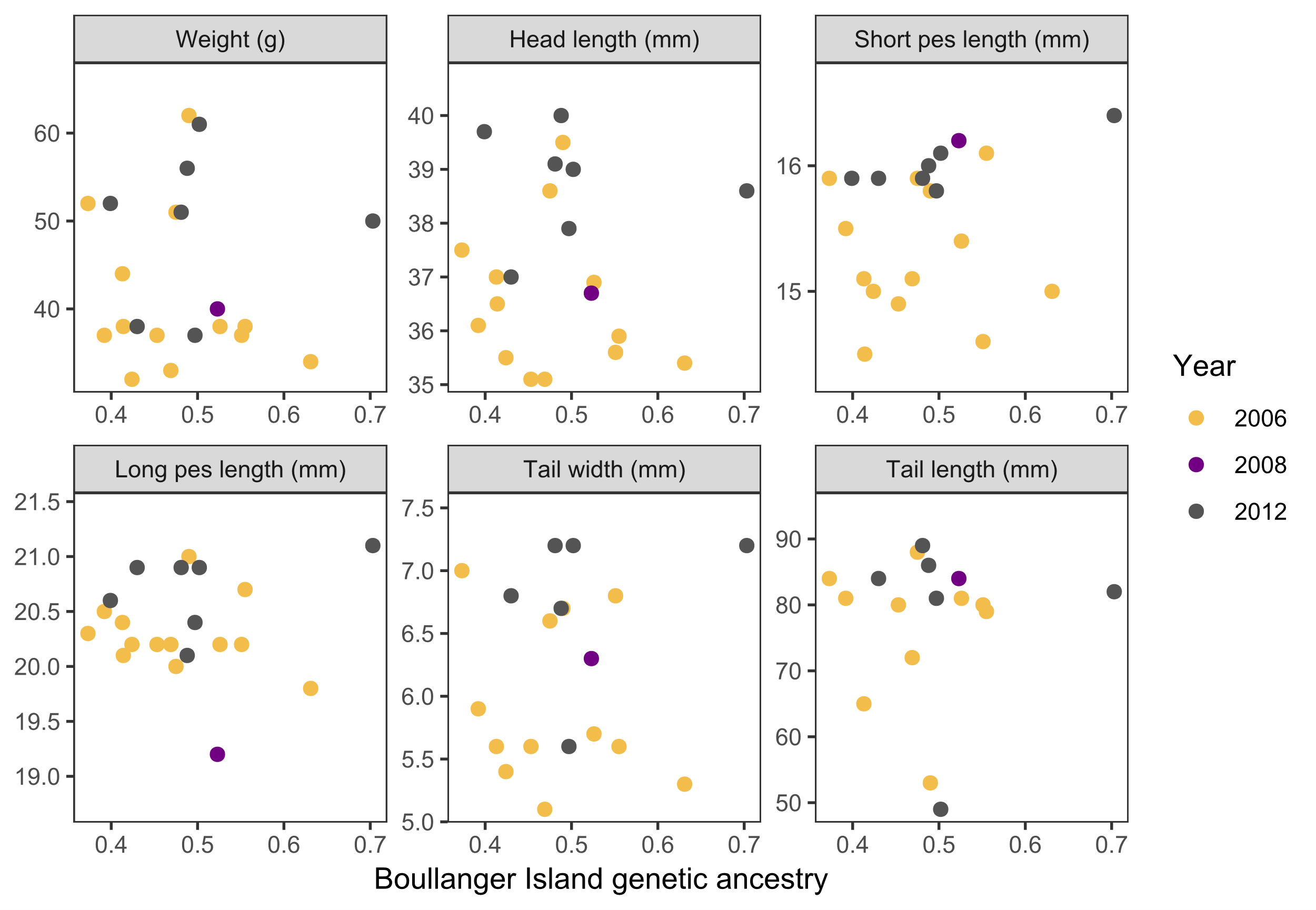
*

**Figure S2** The relationship between Boullanger Island ancestral genetic proportions and body measurement of adult female *P. apicalis* captured on Escape Island between 2006 and 2012.

*
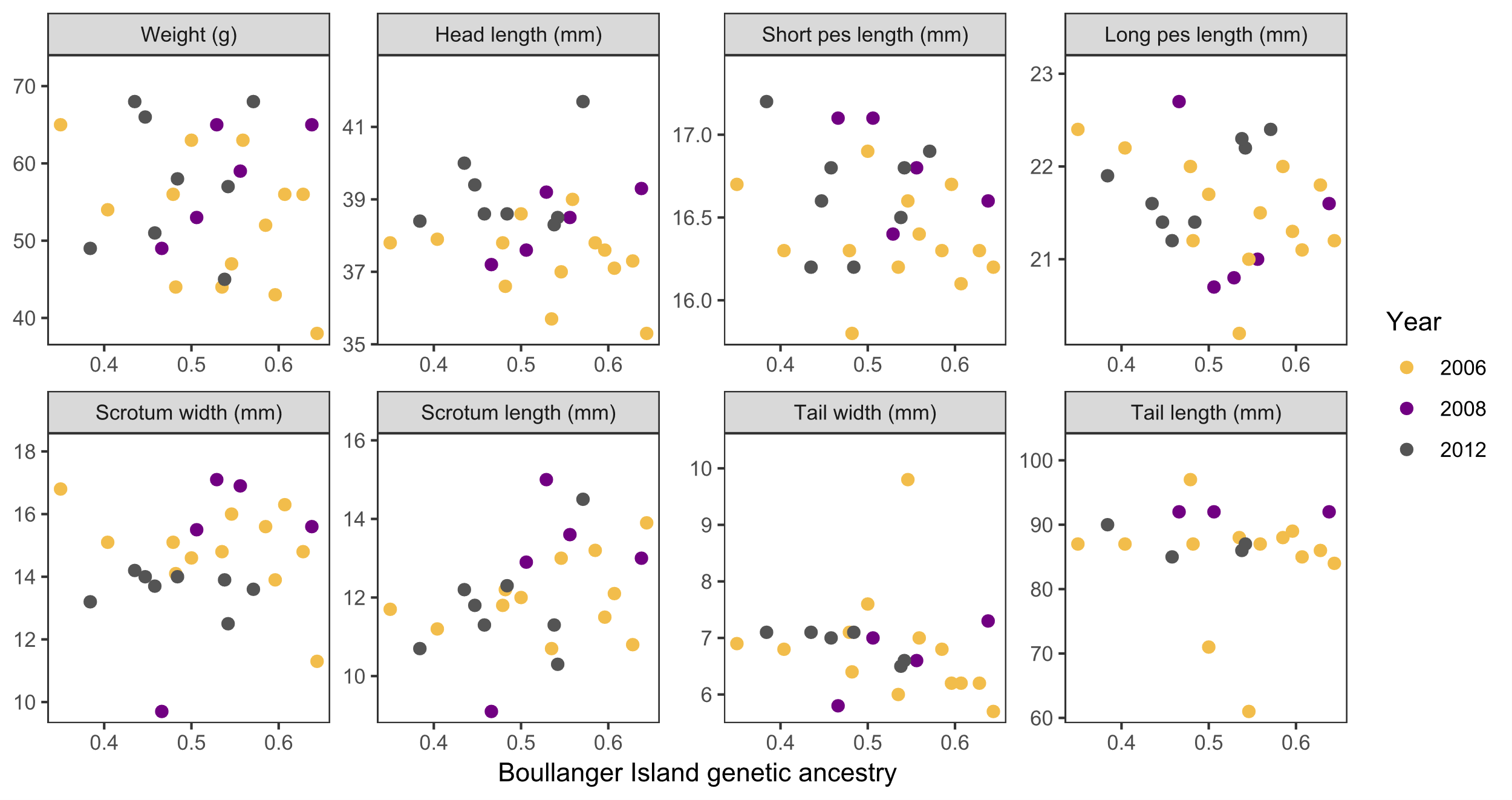
*

**Figure S3** The relationship between Boullanger Island ancestral genetic proportions and body measurement of adult male *P. apicalis* captured on Escape Island between 2006 and 2012.

**Figure S4** Number of pouch young carried by *P. apicalis* females on Boullanger and Whitlock Islands.


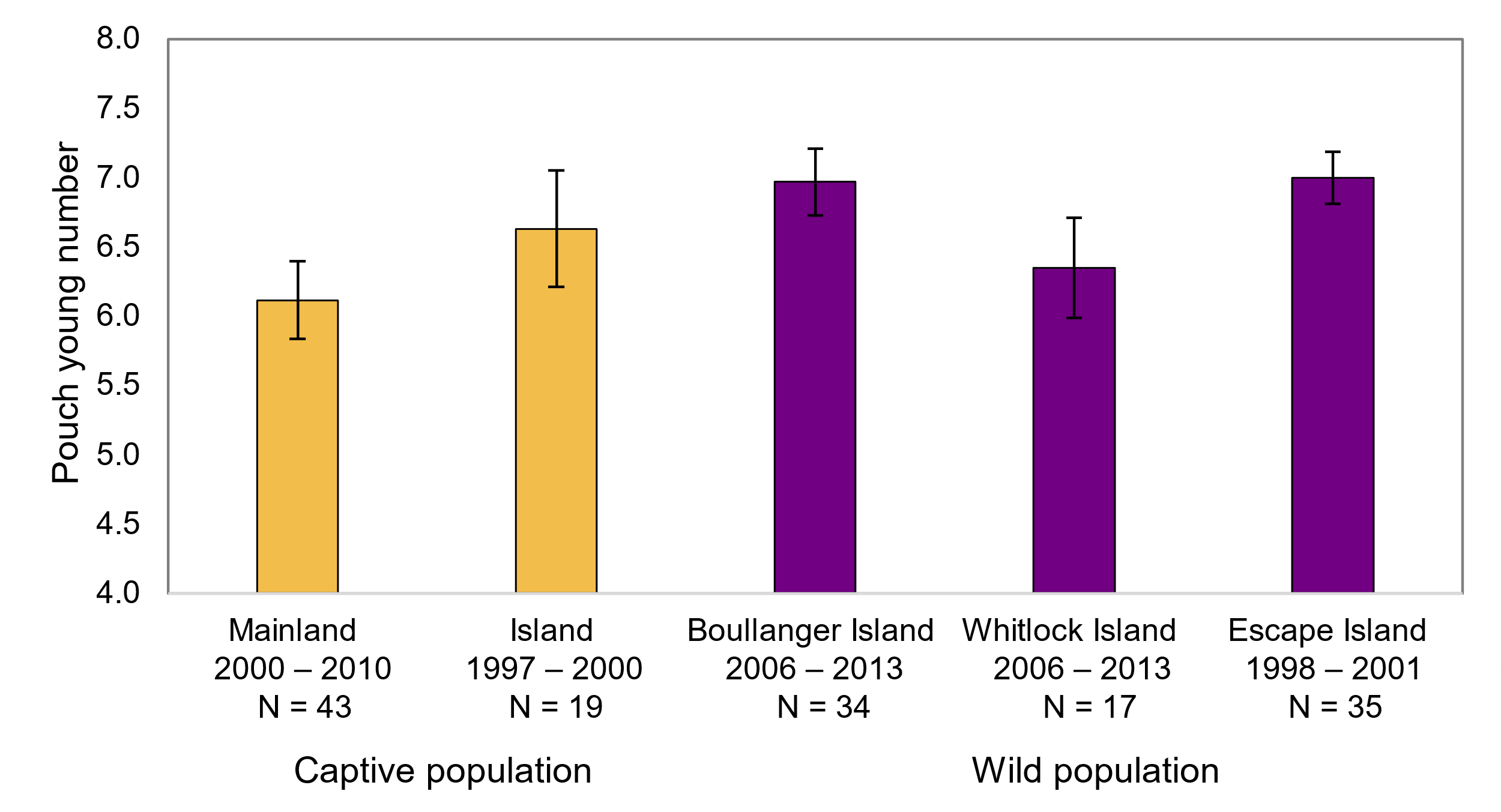


**Figure S5** Average number of pouch young of captive and wild *P. apicalis* populations with years of observation. Captive mainland population and Escape Island observations were referenced from [Thavornkanlapachai et al. (2021)](#_ENREF_8) and [Moro (2003)](#_ENREF_6) respectively. The captive mainland population had observations from 2004 – 2006 removed as the numbers of pouch young were unusually low in all females. The low pouch young numbers may be caused by mothers’ stress in captivity rather than biological variation. N is the number of litters. Error bars are standard errors.

**References**

BANKS, S. C., FINLAYSON, G. R., LAWSON, S. J., LINDENMAYER, D. B., PAETKAU, D., WARD, S. J. & TAYLOR, A. C. 2005. The effects of habitat fragmentation due to forestry plantation establishment on the demography and genetic variation of a marsupial carnivore, *Antechinus agilis*. *Biological Conservation,* 122**,** 581-597.

FIRESTONE, K. B. 1999. Isolation and characterization of microsatellites from carnivorous marsupials (Dasyuridae: Marsupialia). *Molecular Ecology,* 8**,** 1084-1086.

JONES, M. E., PAETKAU, D., GEFFEN, E. & MORITZ, C. 2003. Microsatellites for the Tasmanian devil (*Sarcophilus laniarius*). *Molecular Ecology Notes,* 3**,** 277-279.

KRAAIJEVELD-SMIT, F. J. L., WARD, S. J., TEMPLE-SMITH, P. D. & PAETKAU, D. 2002. Factors influencing paternity success in *Antechinus agilis*: last-male sperm precedence, timing of mating and genetic compatibility. *Journal of Evolutionary Biology,* 15**,** 100-107.

MILLS, H. R. & SPENCER, P. B. S. 2003. Polymorphic microsatellites identified in an endangered dasyurid marsupial, the dibbler (*Parantechinus apicalis*). *Molecular Ecology Notes,* 3**,** 218-220.

MORO, D. 2003. Translocation of captive-bred dibblers *Parantechinus apicalis* (Marsupialia: Dasyuridae) to Escape Island, Western Australia. *Biological Conservation,* 111**,** 305-315.

SPENCER, P. B. S., CARDOSO, M., HOW, R. A., WILLIAMS, J., BUNCE, M. & SCHMITT, L. H. 2007. Cross-species amplification at microsatellite loci in Australian quolls including the description of five new markers from the Chuditch (*Dasyurus geoffroii*). *Molecular Ecology Notes,* 7**,** 1100-1103.

THAVORNKANLAPACHAI, R., MILLS, H., OTTEWELL, K., FRIEND, J. & KENNINGTON, W. 2021. Temporal variation in the genetic composition of an endangered marsupial reflects reintroduction history. *Diversity,* 13**,** 257.
